# Comment on “Panniculectomy Combined with Bariatric Surgery by Laparotomy: An Analysis of 325 Cases”

**DOI:** 10.1155/2016/9709283

**Published:** 2016-01-04

**Authors:** R. Vilallonga

**Affiliations:** Universitary Hospital Vall Hebron, Universitat Autònoma de Barcelona, Pg de la Vall Hebron 119-129, 08035 Barcelona, Spain

We would be happy to comment on the study by Dr. Colabianchi et al. on the role of synchronic panniculectomy and bariatric surgery when performed by laparotomy. As the authors mention in the article, bariatric surgery can be combined with panniculectomy during the first stage. Although controversial, the authors present the experience of 325 cases [1].

Interestingly, there are few reports that mention this approach compared to performing a regular panniculectomy separately from bariatric surgery and only after the weight loss of the patient has been stabilized. This last approach could be better in order to perform an optimal and maximal panniculectomy with a better esthetical result. However, most of the patients will not reach a perfect aesthetic result. In fact, it is not the goal for bariatric surgery. The goal is improving quality of life and reducing the comorbidities associated with the severe obesity [2]. For this purpose, the initial panniculectomy can be more interesting avoiding a second surgery to the patient, with acceptable final results and leading to a better quality of life since the early beginning after bariatric surgery [3].

As we have read, the authors do not report any special complication in the postoperative period once the bariatric surgery is combined with the panniculectomy specially when the technique is perfectly reproduced. Thus, it can be encouraged as far as the procedure is performed open. A clear way to perform the technique, including a specific training or learning curve, should however be included in the protocol before attempting massive panniculectomy to patients simultaneously with bariatric surgery.

Another comment that appears is regarding the approach to perform the bariatric surgery. Interestingly, the authors mention a series belonging to some years ago. Regarding the role of laparoscopy nowadays, the index of penetration of laparoscopy in bariatric surgery has increased in a tremendous way much more even compared to colorectal surgery [4]. This surgical approach would suggest that the panniculectomy cannot be done in the same way, but this still should be demonstrated. When we think of minimally invasive surgery, we do not see a maximal panniculectomy; however, a special focus should be done while differentiating the two procedures although some of the benefits for the laparoscopic surgery in terms of pain control, hospital stay, or wound complications will not be achieved. As the readers know, a cholecystectomy can be performed laparoscopically on a second surgery combined with a panniculectomy after the weight loss of the patient has been stabilized.

More rarely, but not uncommon, a combined panniculectomy during the bariatric laparoscopic surgery has been performed in order to allow pneumoperitoneum and performing the procedure. This combined approach is sometimes necessary to perform a laparoscopic approach.

As the authors mention, further studies combining panniculectomy with laparoscopic bariatric surgery are necessary for comparison of results obtained by the different techniques. We would like to encourage multidisciplinary teams to analyse the benefit of the combined procedure including immediate reduction of the abdominal apron, prevention of lymphedema of the abdominal wall, improved personal hygiene and quality of life and self-esteem, satisfactory results with no need for revision surgery, and weight loss compared to standard laparoscopic procedure with associated panniculectomy after the weight loss of the patient. A very interesting data would be to combine in a randomized control trial patients who undergo bariatric surgery alone and combined and evaluate these issues according to grades, according to Igwe Jr. et al. [5]. More interesting is to know whether it is important to estimate the degree of satisfaction before and after any type of the proposed treatment. There is a clear need for the appropriate management of expectations before surgery is mandatory [6].

We believe that any intervention related to an obese patient must be optimal. The costs and benefits of any intervention should be stressed and it is important to define which is more cost effective the one-stage or two-stage procedure according to the final results and perioperative complications, even combining laparoscopic approach [7].

We can conclude that bariatric surgery can be combined with abdominal panniculectomy; however, a clear and accurate technique is needed in order to improve the patient's quality of life soon after surgery and during the weight loss period.
